# Causal effect of vascular endothelial growth factor on the risk of atrial fibrillation: a two-sample Mendelian randomization study

**DOI:** 10.3389/fcvm.2024.1416412

**Published:** 2024-10-18

**Authors:** Siliang Han, Ling Xue, Chunhong Chen, Junmin Xie, Fanchang Kong, Fang Zhang

**Affiliations:** ^1^ Department of Cardiology, The Second Hospital of Hebei Medical University, Shijiazhuang, Hebei, China; ^2^ Department of Cardiology, Affiliated Hospital of Hebei University, Baoding, Hebei, China

**Keywords:** atrial fibrillation, causal association, Mendelian randomization, vascular endothelial growth factor, VEGF-D

## Abstract

**Background:**

Observational studies have found that vascular endothelial growth factor (VEGF) levels are associated with the risk of cardiovascular disease. However, it remains unclear whether VEGF levels have a causal effect on the risk of atrial fibrillation.

**Methods:**

A two-sample Mendelian randomization (MR) study was conducted to explore the causal relationship between VEGF levels and the risk of atrial fibrillation. Genetic variants associated with VEGF [VEGF-A, VEGF-C, VEGF-D, VEGF receptor−2 (VEGFR-2), VEGFR-3] and atrial fibrillation (atrial fibrillation, atrial fibrillation and flutter) were used as instrumental variables. Data on genetic variants were obtained from published genome-wide association studies (GWAS) or the IEU Open GWAS project. Inverse-variance weighted (IVW) analysis was used as the primary basis for the results, and sensitivity analyses were used to reduce bias. Causal relationships were expressed as odds ratio (OR) with 95% confidence interval (CI), and a *P*-value of <0.1 corrected for False Discovery Rate (FDR) (*P_FDR_* < 0.1) was considered to have a significant causal relationship.

**Results:**

Genetically predicted high levels of VEGF-A [OR = 1.025 (95%CI: 1.004–1.047), *P_FDR_* = 0.060] and VEGF-D [OR = 1.080 (95%CI: 1.039–1.123), *P_FDR_* = 0.001]] were associated with an increased risk of atrial fibrillation, while no causal relationship was observed between VEGF-C (*P_FDR_* = 0.419), VEGFR-2 (*P_FDR_* = 0.784), and VEGFR-3 (*P_FDR_* = 0.899) and atrial fibrillation risk. Moreover, only genetically predicted high levels of VEGF-D [OR = 1.071 (95%CI: 1.014–1.132), *P_FDR_* = 0.087] increased the risk of atrial fibrillation and flutter. Sensitivity analysis demonstrated that the relationship between VEGF-D levels and the risk of atrial fibrillation was robust.

**Conclusion:**

This study supports a causal association between high VEGF-D levels and increased risk of atrial fibrillation.

## Introduction

Atrial fibrillation is the most common arrhythmia and is associated with an increased risk of death, stroke, and peripheral embolism (1). Atrial fibrillation causes a higher disease burden and the prevalence and incidence of atrial fibrillation increases with age (2, 3). Common risk factors for atrial fibrillation are age, female, smoking, alcohol consumption, body mass index, hypertension, atrial fibrosis, left ventricular hypertrophy, heart failure, and myocardial infarction (1, 4). Endothelial dysfunction, inflammation, and oxidative stress play an important role in the pathophysiological process of atrial fibrillation (5–7).

Vascular endothelial growth factor (VEGF) is a class of neurotrophic and angiogenic factors secreted by endothelial cells (8). A cohort study showed that the risk of atrial fibrillation increased with elevated VEGF-D levels (9). Another cohort study found a U-shaped association between VEGF and the risk of cardiovascular events (10). In addition, several studies have shown higher VEGF levels in patients with atrial fibrillation compared to control populations without atrial fibrillation (5, 11). The potential association of VEGF with atrial fibrillation may be related to the role of VEGF as an inflammatory and pro-fibrotic mediator and its involvement in myocardial remodeling (12, 13). However, these observational studies of the association between VEGF and atrial fibrillation cannot infer causality due to confounding factors. Mendelian randomization (MR) uses genetic variation related to exposure to assess causal effects on outcomes (14, 15). Compared to traditional observational studies, MR is less susceptible to measurement error, confounders, and reverse causation, and reflects the long-term effects of exposure on outcomes. Recent MR studies have found a positive association between VEGF levels and the risk of venous thromboembolism (16) and cardiovascular death (17), but no association between VEGF levels and ischemic heart disease (18). However, the causal association between VEGF levels and the risk of atrial fibrillation remains unclear. Thus, the purpose of this study was to assess whether VEGF levels have a causal effect on the risk of atrial fibrillation through a two-sample MR study.

## Methods

### Study design

**Figure 1:**
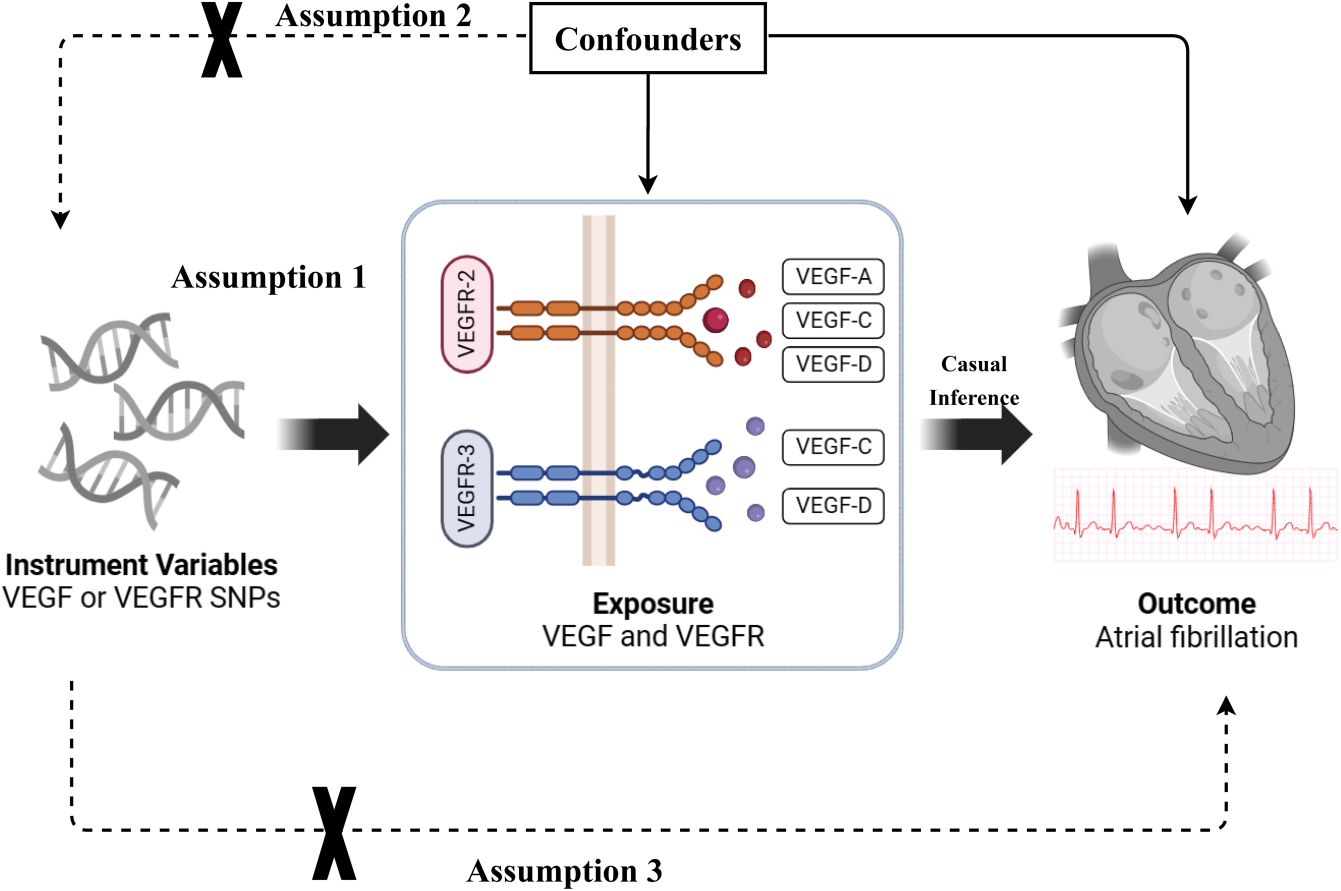
The basic assumptions of Mendelian randomization (MR) study. VEGF, vascular endothelial growth factor; VEGFR, vascular endothelial growth factor receptor; SNP, single nucleotide polymorphism.

The causal association between VEGF levels and atrial fibrillation was examined through a two-sample MR analysis. The study methods were compliant with the STROBE-MR checklist (19). The genetic variants [single nucleotide polymorphisms (SNPs)] significantly associated with VEGF and atrial fibrillation were selected as instrumental variables, respectively. SNPs used for MR analysis should fulfill three basic assumptions: (1) SNPs are strongly associated with exposure; (2) SNPs are not associated with confounders; and (3) SNPs affect the outcomes only through exposure (Figure 1). This study was a secondary analysis of publicly available data. Ethical approval and informed consent were obtained for each of the original studies.

### Selection of instrumental variables

SNPs related to exposure and outcome in this study were obtained from published genome-wide association studies (GWAS) or the IEU Open GWAS project. The IEU Open GWAS project (https://gwas.mrcieu.ac.uk/) is a database of genetic associations from GWAS summary datasets for query or download (20). Table 1 shows the sources of SNPs related to exposure and outcome.

**Table 1:** Data sources on genetic variants associated with exposures and outcomes.

Phenotype	Sample size (Case)	Ancestry	IEU open GWAS ID	PMID
Exposures				
VEGF-A	3,301	European	prot-a-3198	29875488
VEGF-C	3,301	European	prot-a-3199	29875488
VEGF-D	21,758	European	ebi-a-GCST90012069	33067605
VEGF (Uncategorized)	21,758	European	ebi-a-GCST90011995	33067605
VEGFR-2	3,301	European	prot-a-1622	29875488
VEGFR-3	3,301	European	prot-a-1129	29875488
Outcomes				
Atrial fibrillation	588,190 (65,446)	91.4% European	ebi-a-GCST006061	29892015
Atrial fibrillation and flutter	218,792 (22,068)	European	finn-b-I9_AF_EXNONE	–

VEGF, vascular endothelial growth factor; VEGFR, vascular endothelial growth factor receptor.

VEGF was the exposure variable in this study, including VEGF-A, VEGF-C, VEGF-D, VEGF receptor-2 (VEGFR-2), VEGFR-3, and VEGF (uncategorized). SNPs associated with VEGF-A, VEGF-C, VEGFR-2, and VEGFR-3 were obtained from the same GWAS that examined levels of VEGF-A, VEGF-C, VEGFR-2, and VEGFR-3 in 3,301 individuals of European descent in the INTERVAL study (21). The mean age (SD) was 43.6 (14.3) years for INTERVAL subcohort1 and 44.1 (14.2) years for INTERVAL subcohort2. There were 51.6% male in INTERVAL subcohort1 and 49.5% male in INTERVAL subcohort2. According to the GWAS (21), the procedure for testing plasma VEGF-A, VEGF-C, VEGFR-2, and VEGFR-3 is as follows: Blood samples were collected in 6-ml EDTA tubes using standard venepuncture protocols. The tubes were inverted three times and transferred to UK Biocentre (Stockport, UK) at ambient temperature for processing. Plasma was extracted by centrifugation into two 0.8 ml aliquots, which were then stored at −80°C prior to use. The relative concentrations of 3,622 plasma proteins or protein complexes were determined using a multiplexed, aptamer-based approach (SOMAscan assay) utilizing 4,034 modified aptamers.

SNPs related to VEGF-D and VEGF (uncategorized) were derived from a GWAS that examined VEGF-D and VEGF (uncategorized) levels in 21,758 individuals of European descent (22). Information about the population and the VEGF-D and VEGF (uncategorized) measurement process were not presented in the original GWAS.

Atrial fibrillation was the outcome variable in this study, including atrial fibrillation and “atrial fibrillation and flutter”. SNPs associated with atrial fibrillation were obtained from a GWAS on atrial fibrillation that analyzed 65,446 cases and 588,190 referents of 91.4% European ancestry from the Atrial Fibrillation Genetics (AFGen) consortium, the Broad AF Study (Broad AF), and the UK Biobank (UKBB) and the Biobank Japan (BBJ) (23). SNPs related to atrial fibrillation and flutter were derived from the IEU Open GWAS project that analyzed 22,068 cases and 196,724 controls of European ancestry from the FinnGen consortium (https://r7.finngen.fi/). The International Classification of Diseases, 10th Revision (ICD-10) code (I18) was used to identify atrial fibrillation and flutter for this trait, and patients with cardiovascular diseases were was excluded from the control group.

SNPs used for MR analysis are subject to rigorous screening. SNPs associated with exposure were first screened according to the following criteria: (1) SNPs were strongly related to VEGF at the genome-wide significance level threshold (*P* < 5 × 10^−6^); and (2) SNPs with linkage disequilibrium (LD) (r^2^ < 0.01 and clumping distance = 10,00 kb) or SNPs being palindromic with intermediate allele frequencies were excluded. After the screening for exposure-related SNPs was completed, the exposure-related SNPs and the outcome-related SNPs were harmonized so that both had the same allele.

### Statistical analysis

The causal relationship between VEGF levels and the risk of atrial fibrillation was analyzed by a variety of MR methods, including inverse-variance weighted (IVW), weighted-median, MR-Egger, weighted mode, and MR-PRESSO. In this study, IVW analysis (fixed effect) was used as the primary basis for the results, supplemented by other MR analysis methods. IVW can obtain unconfounded estimates of genetically predicted exposure on outcome based on the Wald ratio method, which is the primary analysis for generating estimates of causal effects in MR analyses (24). Weighted-median provides a robust and consistent estimate of the effect even if nearly 50% of genetic variants were invalid instruments (25). MR-Egger provides valid tests and consistent estimates of causal effects even when all instrumental variables are invalid, but MR-Egger may exhibit low precision and be affected by abnormal genetic variation (26). Weighted mode requires that the most common causal effect estimate is a consistent estimate of the true causal effect even if the majority of instruments are invalid (27). MR-PRESSO detects and attempts to reduce horizontal pleiotropy by removing significant outliers, but the MR-PRESSO outlier test requires at least 50% of the genetic variants to be valid instruments and relies on Instrument Strength Independent of Direct Effect (InSIDE) assumptions (28).

For the detection of SNPs for MR analysis, strength, horizontal pleiotropy, and heterogeneity tests were performed. The strength of SNPs was examined using the F-statistic and variance explained (*R*^2^), and SNPs with an F-statistic less than 10 were considered weak instrument variables (29). The *R*^2^-value (30) and F-statistics (29) are calculated as follows:

$$R^{2}=\overset{k}{\underset{i=1}{\sum }}\frac{2\beta_{i}^{2}MAF_{SNPi}(1-MAF_{SNPi})}{SD^{2}}$$

In the formula, MAF is the minor allele frequency, *β* is the effect size of SNP on exposure, SD is the standard deviation, *k* is the number of instrumental variables, and *n* is the sample size of exposure.

$$F=\frac{R^{2}(n-1-k)}{(1-R^{2})k}$$

The existence of horizontal pleiotropy, in which a genetic variant is associated with multiple risk factors on different causal pathways, violates the assumptions underlying MR analysis. The MR-Egger intercept test was used to examine horizontal pleiotropy, and a *P*-value of less than 0.05 for the intercept was considered to have horizontal pleiotropy (26). Heterogeneity was assessed by Cochran's Q statistic for MR-Egger and IVW analyses, with a *P*-value for the Q statistic of less than 0.05 considered to be present (31). For SNPs with heterogeneity, the analysis results were based on the random effects model of IVW. The leave-one-out analysis was utilized to assess whether the causal relationship was driven by an individual SNP. In addition, the bidirectional MR analysis was performed to evaluate whether there was a reverse causal association between VEGF levels and atrial fibrillation. All MR analyses were performed using the “TwoSampleMR” package in R software (version 4.2.3). A *P*-value <0.05 was considered statistically significant and the False Discovery Rate (FDR) was used to correct the *P*-value and an FDR-corrected *P*-value <0.1 (P_FDR_ <0.1) was considered to have a significant causal relationship (32, 33).

## Results

### Characteristics of SNPs for analysis

Table 2 shows the test results of the SNPs used for MR analysis. For the analysis of atrial fibrillation, the number of SNPs used for MR analysis was 19, 13, 27, 19, 15, and 20 for VEGF-A, VEGF-C, VEGF-D, VEGF (uncategorized), VEGFR-2, and VEGFR-3, respectively. The strength test showed that the F-statistic of these included SNPs ranged from 37 to 108, representing that there were no weak instrument variables in these SNPs. The results of the horizontal pleiotropy test showed that the *P*-values of the intercepts of these SNPs were all greater than 0.05, indicating that there was no horizontal pleiotropy for these SNPs. The heterogeneity test demonstrated that there was no heterogeneity in the analysis between VEGF and atrial fibrillation and flutter (*P* > 0.05), but there was heterogeneity in the analysis between VEGF-C and atrial fibrillation as well as between VEGF (uncategorized) and atrial fibrillation (*P* < 0.05).

**Table 2:** Test results of the SNPs used for MR analysis.

Variables	Selected SNP (*P* < 5E-6)	Omitted LD SNP	Drop all palindromic	Horizontal pleiotropy test		Heterogeneity test				Strength		MR-PRESSO Outlier SNP
				MR-Egger intercept	*P*	MR Egger Q- statistic	*P*	IVW Q- statistic	*P*	*F*-value	*R^2^*(%)	
Atrial fibrillation												
VEGF-A	174	20	19	4.72E-04	0.915	15.48	0.561	15.49	0.628	40	1.15	–
VEGF-C	94	16	13	−0.008	0.248	20.60	0.038	23.40	0.025	38	1.11	rs41278571
VEGF-D	731	29	27	−8.93E-05	0.984	22.32	0.617	22.32	0.671	37	0.17	–
VEGF (Uncategorized)	677	19	19	0.008	0.190	50.22	3.91E-05	55.72	9.88E-06	84	0.38	rs10822155
VEGFR-2	505	16	15	−4.43E-04	0.938	17.29	0.187	17.29	0.241	51	1.48	–
VEGFR-3	1,402	23	20	0.004	0.275	22.20	0.223	23.77	0.205	77	1.95	–
Atrial fibrillation and flutter												
VEGF-A	174	20	20	−0.006	0.450	19.42	0.367	20.06	0.391	39	1.13	–
VEGF-C	94	16	13	−0.013	0.091	14.50	0.207	19.03	0.088	38	1.11	–
VEGF-D	731	29	27	0.002	0.750	24.11	0.513	24.22	0.564	37	0.17	–
VEGF (Uncategorized)	677	19	19	0.006	0.392	20.50	0.250	21.42	0.259	84	0.38	–
VEGFR-2	505	16	16	−0.005	0.491	12.51	0.565	13.01	0.601	72	2.03	–
VEGFR-3	1,402	23	22	0.010	0.184	27.12	0.132	29.69	0.098	89	2.27	–

SNP, single nucleotide polymorphism; MR, mendelian randomization; VEGF, vascular endothelial growth factor; VEGFR, vascular endothelial growth factor receptor; LD, linkage disequilibrium; IVW, inverse variance weighted; MR-PRESSO, MR Pleiotropy RESidual Sum and Outlier tes.

### Causal effect of VEGF on the risk of atrial fibrillation

**Figure 2:**
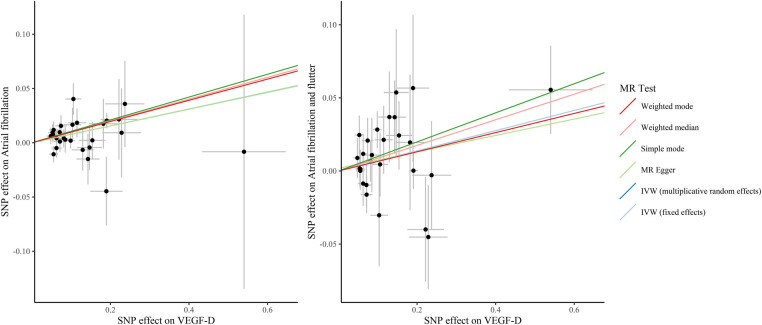
The scatter plots of the association between VEGF-D and the risk of atrial fibrillation and atrial fibrillation and flutter. VEGF, vascular endothelial growth factor; SNP, single nucleotide polymorphism; IVW, inverse variance weighted; MR, Mendelian randomization.

**Figure 3:**
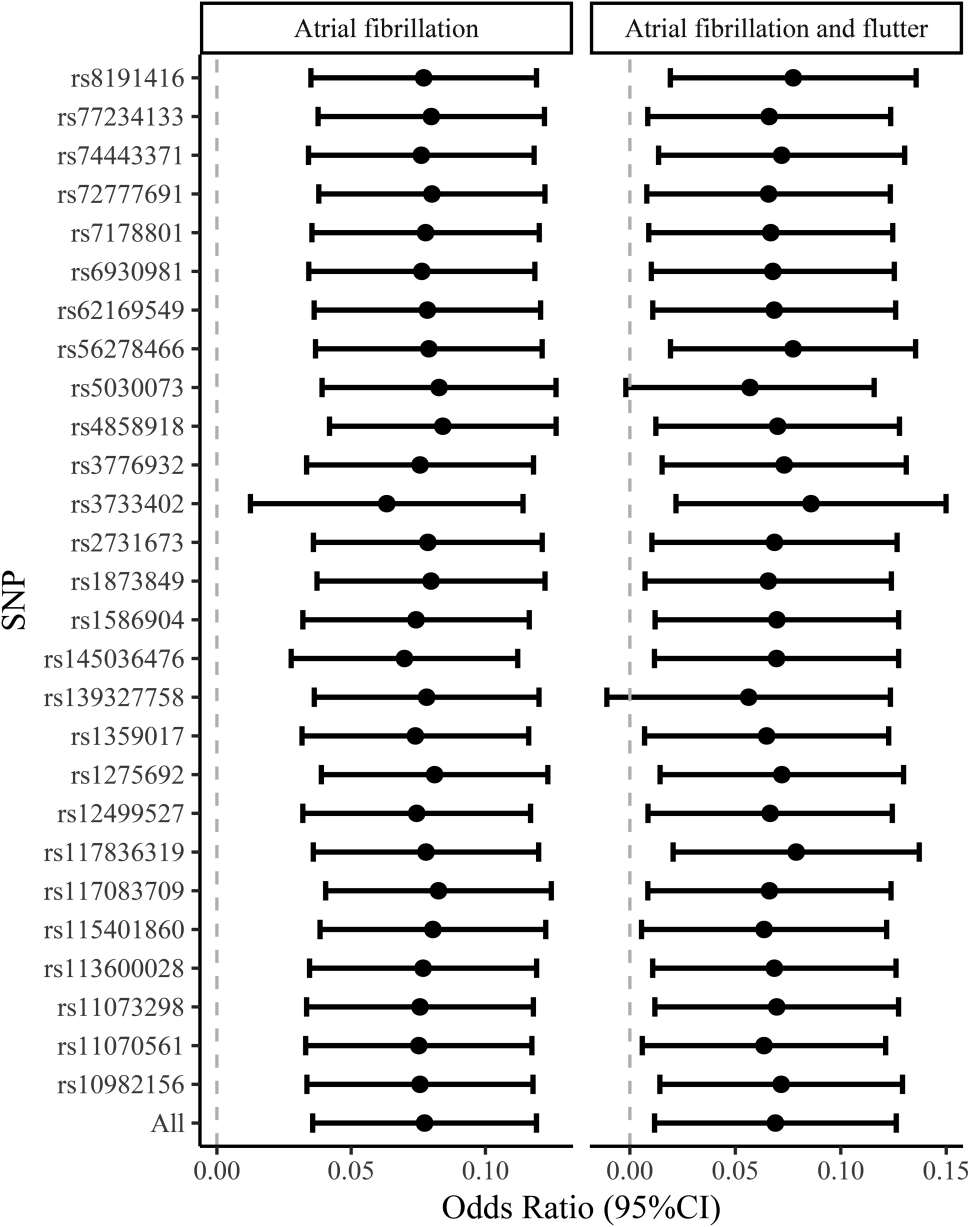
The leave-one-out analysis of the causal association between VEGF-D and the risk of atrial fibrillation and atrial fibrillation and flutter. VEGF, vascular endothelial growth factor.

Table 3 presents the IVW results for the causal relationship between VEGF levels and the risk of atrial fibrillation. In the analysis of atrial fibrillation, genetically predicted high levels of VEGF-A [IVW: odds ratio (OR) = 1.025 (95%CI: 1.004–1.047), *P* = 0.020, *P_FDR_* = 0.060] and VEGF-D [IVW: OR = 1.080 (95%CI: 1.039–1.123), *P* = 8.66E-05, *P_FDR_* = 0.001] were associated with an increased risk of atrial fibrillation, while no causal relationship was observed between VEGF-C (*P_FDR_* = 0.419), VEGF (uncategorized) (*P_FDR_* = 0.383), VEGFR-2 (*P_FDR_* = 0.784), and VEGFR-3 (*P_FDR_* = 0.899) and atrial fibrillation risk. In the analysis of atrial fibrillation and flutter, only genetically predicted high levels of VEGF-D [IVW: OR = 1.071 (95%CI: 1.014–1.132), *P* = 0.014, *P_FDR_* = 0.087] increased the risk of atrial fibrillation and flutter. The supplement results of weighted-median, MR-Egger, weighted mode, and MR-PRESSO analyses of the association between VEGF and atrial fibrillation risk were shown in Supplementary Table S1. The direction of the association between VEGF-A and atrial fibrillation, between VEGF-D and atrial fibrillation, and between VEGF-D and atrial fibrillation and flutter results in weighted-median, MR-Egger, weighted mode, and MR-PRESSO analyses remained consistent with IVW. The effect of VEGF on atrial fibrillation in these supplementary analyses was consistent with the results of the IVW analysis. The scatter plots of the association between VEGF-D and the risk of atrial fibrillation and atrial fibrillation and flutter were presented in Figure 2. The leave-one-out analysis demonstrated that the causal association between VEGF-D and the risk of atrial fibrillation and atrial fibrillation and flutter was robust (Figure 3). In addition, the bidirectional MR analysis showed no reverse causal association between VEGF levels and atrial fibrillation (Supplementary Table S2).

**Table 3:** The IVW results for the causal relationship between VEGF levels and the risk of atrial fibrillation.

Variables	Methods	SNPs (*n*)	Atrial fibrillation			SNPs (*n*)	Atrial fibrillation and flutter		
			OR (95%CI)	*P*	*P_FDR_*		OR (95%CI)	*P*	*P_FDR_*
VEGF-A	IVW (RE)	19	1.025 (1.004–1.047)	0.020	0.060	20	0.977 (0.942–1.013)	0.204	0.245
	IVW (FE)		1.025 (1.002–1.048)	0.031	0.062		0.977 (0.943–1.012)	0.192	0.230
VEGF-C	IVW (RE)	13	0.977 (0.938–1.019)	0.279	0.419	16	1.002 (0.969–1.036)	0.917	0.917
	IVW (FE)		0.977 (0.949–1.007)	0.131	0.196		1.002 (0.975–1.029)	0.895	0.895
VEGF-D	IVW (RE)	27	1.080 (1.039–1.123)	8.66E-05	0.001	29	1.071 (1.014–1.132)	0.014	0.087
	IVW (FE)		1.080 (1.036–1.126)	2.76E-04	0.002		1.071 (1.012–1.135)	0.018	0.110
VEGF (Uncategorized)	IVW (RE)	19	1.031 (0.985–1.079)	0.191	0.383	19	1.033 (0.986–1.082)	0.176	0.245
	IVW (FE)		1.031 (1.005–1.058)	0.022	0.062		1.033 (0.989–1.078)	0.140	0.210
VEGFR-2	IVW (RE)	15	0.994 (0.969–1.020)	0.654	0.784	16	0.973 (0.947–1.000)	0.052	0.156
	IVW (FE)		0.994 (0.971–1.018)	0.618	0.741		0.973 (0.945–1.002)	0.070	0.141
VEGFR-3	IVW (RE)	20	0.999 (0.981–1.017)	0.899	0.899	22	0.978 (0.950–1.006)	0.125	0.245
	IVW (FE)		0.999 (0.983–1.015)	0.887	0.887		0.978 (0.954–1.002)	0.068	0.141

IVW, inverse variance weighted; RE, random effect model; FE, fixed effect model; FDR, False Discovery Rate; VEGF, vascular endothelial growth factor; VEGFR, vascular endothelial growth factor receptor; SNP, single nucleotide polymorphism; OR, odds ratio; CI, confidence interval.

## Discussion

This study assessed the causal relationship between VEGF levels and the risk of atrial fibrillation using MR analysis. The results demonstrated that genetically predicted high levels of VEGF-D increased the risk of atrial fibrillation and atrial fibrillation and flutter. Sensitivity analysis showed that the causal relationship between VEGF-D levels and the risk of atrial fibrillation was robust.

Stretch fibrosis, epicardial adipose tissue, inflammation, autonomic nervous system imbalance, and genetic mutations have been associated with the development of atrial fibrillation (34). The VEGF family are regulators of blood and lymph vessel formation (8). Several studies have found an association between VEGF levels and cardiovascular disease risk (9, 10, 18). An observational study found that elevated VEGF-D levels were associated with an increased risk of atrial fibrillation and ischemic stroke (9). However, observational studies are susceptible to a variety of confounding factors that prevent inferences of causality. Thus, our study investigated whether VEGF levels have a causal effect on the risk of atrial fibrillation. Among the VEGF types analyzed in our study, VEGF-A mainly regulates vascular growth, and VEGF-C and VEGF-D mainly regulate lymphangiogenesis. For VEGF receptors (VEGFR), VEGFR-2 mainly promotes angiogenesis expressed in vascular endothelial cells, and VEGFR-3 mainly promotes lymphogenesis expressed in lymphatic endothelial cells, in which VEGF-A binds to VEGFR-2, while VEGF-C and VEGF-D bind to VEGFR-2 and VEGFR-3 (8). Our results found that genetically predicted high levels of VEGF-D increased the risk of atrial fibrillation. Our findings were consistent with previous observational studies in which elevated AEGF-D levels increased the risk of atrial fibrillation. However, we did not find a causal association between VEGFR-2 and VEGFR-3 levels and the risk of atrial fibrillation. VEGF is involved in a variety of functions in the body such as the promotion of angiogenesis and lymphogenesis, regulation of inflammation, resistance to oxidative stress, fibrosis, and regulation of lipid metabolism (35). The regulation of these functions may be involved in the development of atrial fibrillation.

VEGF-D has been reported to regulate lymphangiogenesis, angiogenesis, and endothelial proliferation via VEGFR-2 or VEGFR-3 (13, 36). The role of VEGF-D and its receptor VEGFR-3 in central pathways for lymphatic vessel development, growth, and maintenance may affect the regulation of interstitial fluid balance and inflammation (37). In addition, VEGF-D may promote cardiac fibrosis by stimulating myofibroblast growth, migration, and collagen synthesis, thereby regulating cardiac repair and remodeling (13). Atrial fibrosis is an important pathophysiological factor in atrial fibrillation and is associated with atrial fibrillation recurrence and complications (38). Elevated VEGF-D levels increase the risk of atrial fibrillation, and higher VEGF-D levels are also observed in patients with atrial fibrillation than in the control population (10). The pulmonary veins are an important source of ectopic beats that trigger atrial tachycardia and atrial fibrillation and maintain atrial fibrillation (39, 40). Atrial fibrillation leads to irregular blood flow, which may cause pulsatile vascular stretching and impaired blood rheology, thereby triggering VEGF secretion from cardiomyocytes and pulmonary vein smooth muscle, and consequently higher VEGF levels are observed in patients with atrial fibrillation (11, 41). However, the biological mechanisms by which elevated VEGF-D levels contribute to the increased risk of atrial fibrillation may need to be further investigated.

The current study explored the causal relationship between VEGF levels and VEGFR levels and the risk of atrial fibrillation using MR analysis. Although the MR study design is less susceptible to potential confounders and inverse causality, several limitations of this study should be considered. First, the GWAS summary data used in this study were obtained from the European population, and the findings may not be generalizable to other ethnic populations. Second, the SNP data related to atrial fibrillation included a small number of individuals from non-European ancestry (nearly 9%), which could have biased the atrial fibrillation-related results. However, we further confirmed the reliability of the association between atrial fibrillation and VEGF levels using atrial fibrillation and flutter data from the FinnGen database. Third, causal associations between VEGF levels and different types of atrial fibrillation (paroxysmal, persistent) could not be analyzed due to a lack of data. Fourth, the lack of individual raw data prevents further exploration of the non-linear association between VEGF levels and the risk of atrial fibrillation. Fifth, the small sample size of the population associated with VEGF in the original GWAS makes the instrumental variables for some of the exposures less well explained. Sixth, there was a 0.32% sample overlap when analyzing the association of VEGF-D and VEGF (Uncategorized) with atrial fibrillation (Supplementary Table S3), which may have affected the relevant results.

## Conclusions

For the different types of VEGF (VEGF-A, VEGF-C, VEGF-D) and VEGFR (VEGFR-2, VEGFR-3), only genetically predicted high levels of VEGF-D increased the risk of atrial fibrillation. The causal association between VEGF levels and the risk of different types of atrial fibrillation and the biological mechanism of the effect of VEGF-D levels on the risk of atrial fibrillation may require further investigation.

## Data Availability

The datasets presented in this study can be found in online repositories. The names of the repository/repositories and accession number(s) can be found in the article/Supplementary Material.
