# Supplementary material for: Causal effect of vascular endothelial growth factor on the risk of atrial fibrillation: a two-sample Mendelian randomization study
Source: Front Cardiovasc Med. 2024 Oct 18;11:1416412. doi: 10.3389/fcvm.2024.1416412 (PMC11527688; doi:10.3389/fcvm.2024.1416412)
Supplement: Supplementary file 1 [file Table1.docx]

**Supplement Table 1**. The results of weighted-median, MR-Egger, weighted mode, and MR-PRESSO analyses of the association between VEGF and atrial fibrillation risk.

| **Variables** | **Methods** | **Atrial fibrillation** | | | **Atrial fibrillation and flutter** | | |
| --- | --- | --- | --- | --- | --- | --- | --- |
|  |  | OR (95%CI) | *P* | *P_FDR_* | OR (95%CI) | *P* | *P_FDR_* |
| VEGF-A | MR Egger | 1.026 (0.980-1.073) | 0.288 | 0.710 | 1.001 (0.932-1.075) | 0.985 | 0.985 |
|  | Weighted median | 1.019 (0.988-1.051) | 0.238 | 0.511 | 0.999 (0.950-1.051) | 0.968 | 0.968 |
|  | Weighted mode | 1.018 (0.987-1.050) | 0.266 | 0.655 | 0.996 (0.945-1.049) | 0.881 | 0.885 |
|  | MR-PRESSO | 1.025 (1.004-1.047) | 0.032 | - | 0.977 (0.942-1.013) | 0.220 | - |
| VEGF-C | MR Egger | 1.019 (0.964-1.076) | 0.530 | 0.894 | 1.023 (0.985-1.062) | 0.269 | 0.537 |
|  | Weighted median | 1.002 (0.959-1.046) | 0.943 | 0.744 | 1.010 (0.981-1.040) | 0.516 | 0.791 |
|  | Weighted mode | 1.018 (0.974-1.064) | 0.449 | 0.688 | 1.011 (0.981-1.043) | 0.474 | 0.664 |
|  | MR-PRESSO | 0.977 (0.938-1.019) | 0.301 | - | 1.002 (0.969-1.036) | 0.919 | - |
|  | MR-PRESSO (Outlier corrected) | 0.947 (0.905-0.991) | 0.038 | - | - | - | - |
| VEGF-D | MR Egger | 1.084 (0.988-1.189) | 0.104 | 0.624 | 1.058 (0.960-1.166) | 0.268 | 0.537 |
|  | Weighted median | 1.105 (1.038-1.176) | 0.002 | 0.011 | 1.092 (1.003-1.188) | 0.043 | 0.165 |
|  | Weighted mode | 1.103 (1.032-1.180) | 0.009 | 0.055 | 1.068 (0.975-1.169) | 0.167 | 0.363 |
|  | MR-PRESSO | 1.080 (1.039-1.123) | 0.001 | - | 1.071 (1.014-1.132) | 0.022 | - |
| VEGF (Uncategorized) | MR Egger | 0.995 (0.930-1.065) | 0.894 | 0.894 | 1.008 (0.938-1.083) | 0.834 | 0.985 |
|  | Weighted median | 1.018 (0.987-1.050) | 0.257 | 0.511 | 1.009 (0.959-1.062) | 0.717 | 0.864 |
|  | Weighted mode | 1.018 (0.988-1.049) | 0.253 | 0.655 | 1.015 (0.964-1.069) | 0.572 | 0.683 |
|  | MR-PRESSO | 1.031 (0.985-1.079) | 0.208 | - | 1.033 (0.986-1.082) | 0.193 | - |
|  | MR-PRESSO (Outlier corrected) | 1.019 (0.993-1.045) | 0.166 | - | - | - | - |
| VEGFR-2 | MR Egger | 0.996 (0.951-1.042) | 0.851 | 0.894 | 0.986 (0.941-1.034) | 0.579 | 0.869 |
|  | Weighted median | 0.994 (0.963-1.027) | 0.731 | 0.744 | 0.963 (0.924-1.005) | 0.082 | 0.165 |
|  | Weighted mode | 0.993 (0.960-1.028) | 0.707 | 0.688 | 0.965 (0.923-1.009) | 0.137 | 0.363 |
|  | MR-PRESSO | 0.994 (0.969-1.020) | 0.660 | - | 0.973 (0.947-1.000) | 0.071 | - |
| VEGFR-3 | MR Egger | 0.986 (0.958-1.015) | 0.355 | 0.710 | 0.954 (0.912-0.998) | 0.053 | 0.320 |
|  | Weighted median | 0.994 (0.975-1.013) | 0.541 | 0.744 | 0.969 (0.939-1.001) | 0.058 | 0.165 |
|  | Weighted mode | 0.994 (0.973-1.014) | 0.542 | 0.688 | 0.967 (0.941-0.995) | 0.029 | 0.282 |
|  | MR-PRESSO | 0.999 (0.981-1.017) | 0.900 | - | 0.978 (0.950-1.006) | 0.140 | - |

Note: MR, mendelian randomization; VEGF, vascular endothelial growth factor; VEGFR, vascular endothelial growth factor receptor; FDR, False Discovery Rate; OR, odds ratio; CI, confidence interval.

**Supplement Table 2**. The bidirectional MR analysis of causal relationship between VEGF levels and atrial fibrillation.

| **Outcome** | **Method** | **Atrial fibrillation (exposure)** | | | | **Atrial fibrillation and flutter (exposure)** | | | |
| --- | --- | --- | --- | --- | --- | --- | --- | --- | --- |
|  |  | SNPs (n) | *β* (95%*CI*) | *P* | *P_FDR_* | SNPs (n) | *β* (95%*CI*) | *P* | *P_FDR_* |
| VEGF-A | IVW (FE) | 90 | -0.016 (-0.097, 0.065) | 0.695 | 0.695 | 39 | 0.027 (-0.044, 0.098) | 0.458 | 0.695 |
|  | IVW (RE) | 90 | -0.016 (-0.100, 0.068) | 0.706 | 0.706 | 39 | 0.027 (-0.038, 0.091) | 0.412 | 0.706 |
|  | MR Egger | 90 | 0.022 (-0.186, 0.231) | 0.836 | 0.836 | 39 | 0.045 (-0.104, 0.195) | 0.557 | 0.836 |
|  | Simple mode | 90 | -0.113 (-0.369, 0.143) | 0.39 | 0.781 | 39 | 0.025 (-0.170, 0.221) | 0.802 | 0.802 |
|  | Weighted median | 90 | -0.054 (-0.181, 0.074) | 0.409 | 0.409 | 39 | 0.049 (-0.065, 0.163) | 0.403 | 0.409 |
|  | Weighted mode | 90 | -0.074 (-0.241, 0.093) | 0.389 | 0.660 | 39 | 0.029 (-0.099, 0.157) | 0.66 | 0.660 |
|  | MR-PRESSO | 90 | -0.016 (-0.100-0.068) | 0.706 | - | 39 | 0.027 (-0.038-0.091) | 0.418 | - |
| VEGF-C | IVW (FE) | 90 | -0.023 (-0.103, 0.058) | 0.585 | 0.696 | 39 | -0.014 (-0.085, 0.057) | 0.696 | 0.696 |
|  | IVW (RE) | 90 | -0.023 (-0.104, 0.059) | 0.589 | 0.688 | 39 | -0.014 (-0.083, 0.055) | 0.688 | 0.688 |
|  | MR Egger | 90 | -0.100 (-0.302, 0.102) | 0.334 | 0.334 | 39 | 0.135 (-0.014, 0.285) | 0.085 | 0.169 |
|  | Simple mode | 90 | -0.056 (-0.310, 0.198) | 0.665 | 0.785 | 39 | 0.032 (-0.196, 0.260) | 0.785 | 0.785 |
|  | Weighted median | 90 | -0.025 (-0.145, 0.095) | 0.684 | 0.684 | 39 | 0.032 (-0.075, 0.138) | 0.561 | 0.684 |
|  | Weighted mode | 90 | -0.020 (-0.192, 0.151) | 0.817 | 0.817 | 39 | 0.068 (-0.053, 0.188) | 0.28 | 0.560 |
|  | MR-PRESSO | 90 | -0.023 (-0.104-0.059) | 0.590 | - | 39 | -0.014 (-0.083-0.055) | 0.690 | - |
| VEGF-D | IVW (FE) | 90 | 0.020 (-0.016, 0.055) | 0.274 | 0.342 | 41 | 0.015 (-0.016, 0.046) | 0.342 | 0.342 |
|  | IVW (RE) | 90 | 0.020 (-0.019, 0.058) | 0.318 | 0.326 | 41 | 0.015 (-0.015, 0.045) | 0.326 | 0.326 |
|  | MR Egger | 90 | -0.011 (-0.106, 0.083) | 0.817 | 0.817 | 41 | -0.035 (-0.099, 0.029) | 0.286 | 0.572 |
|  | Simple mode | 90 | 0.013 (-0.095, 0.122) | 0.812 | 0.833 | 41 | 0.008 (-0.066, 0.082) | 0.833 | 0.833 |
|  | Weighted median | 90 | 0.014 (-0.039, 0.068) | 0.606 | 0.714 | 41 | -0.009 (-0.055, 0.038) | 0.714 | 0.714 |
|  | Weighted mode | 90 | 0.020 (-0.048, 0.089) | 0.56 | 0.619 | 41 | -0.012 (-0.060, 0.036) | 0.619 | 0.619 |
|  | MR-PRESSO | 90 | 0.020 (-0.019-0.058) | 0.321 | - | 41 | 0.015 (-0.015-0.045) | 0.332 | - |
| VEGF (Uncategorized) | IVW (FE) | 90 | 0.006 (-0.031, 0.043) | 0.756 | 0.965 | 41 | -0.001 (-0.033, 0.032) | 0.965 | 0.965 |
|  | IVW (RE) | 90 | 0.006 (-0.037, 0.049) | 0.787 | 0.966 | 41 | -0.001 (-0.035, 0.033) | 0.966 | 0.966 |
|  | MR Egger | 90 | -0.028 (-0.133, 0.078) | 0.611 | 0.788 | 41 | -0.010 (-0.080, 0.061) | 0.788 | 0.788 |
|  | Simple mode | 90 | 0.001 (-0.130, 0.131) | 0.99 | 0.990 | 41 | 0.008 (-0.093, 0.110) | 0.872 | 0.990 |
|  | Weighted median | 90 | 0.016 (-0.044, 0.076) | 0.605 | 0.882 | 41 | 0.004 (-0.049, 0.057) | 0.882 | 0.882 |
|  | Weighted mode | 90 | 0.032 (-0.067, 0.130) | 0.532 | 0.805 | 41 | 0.007 (-0.045, 0.058) | 0.805 | 0.805 |
|  | MR-PRESSO | 90 | 0.006 (-0.037-0.049) | 0.788 | - | 41 | -0.001 (-0.035-0.033) | 0.967 | - |
| VEGFR-2 | IVW (FE) | 90 | 0.054 (-0.027, 0.135) | 0.189 | 0.189 | 39 | 0.061 (-0.010, 0.132) | 0.092 | 0.185 |
|  | IVW (RE) | 90 | 0.054 (-0.034, 0.143) | 0.23 | 0.230 | 39 | 0.061 (-0.010, 0.132) | 0.091 | 0.181 |
|  | MR Egger | 90 | 0.145 (-0.074, 0.363) | 0.199 | 0.328 | 39 | 0.076 (-0.074, 0.227) | 0.328 | 0.328 |
|  | Simple mode | 90 | 0.065 (-0.206, 0.337) | 0.638 | 0.638 | 39 | 0.094 (-0.112, 0.299) | 0.376 | 0.638 |
|  | Weighted median | 90 | 0.057 (-0.070, 0.184) | 0.382 | 0.419 | 39 | 0.049 (-0.070, 0.167) | 0.419 | 0.419 |
|  | Weighted mode | 90 | 0.049 (-0.130, 0.227) | 0.593 | 0.593 | 39 | 0.043 (-0.077, 0.163) | 0.485 | 0.593 |
|  | MR-PRESSO | 90 | 0.054 (-0.034-0.143) | 0.233 | - | 39 | 0.061 (-0.010-0.132) | 0.099 | - |
| VEGFR-3 | IVW (FE) | 90 | 0.070 (-0.010, 0.151) | 0.088 | 0.176 | 39 | 0.024 (-0.047, 0.096) | 0.503 | 0.503 |
|  | IVW (RE) | 90 | 0.070 (-0.009, 0.150) | 0.081 | 0.163 | 39 | 0.024 (-0.044, 0.093) | 0.486 | 0.486 |
|  | MR Egger | 90 | 0.067 (-0.133, 0.267) | 0.514 | 0.976 | 39 | -0.002 (-0.152, 0.147) | 0.976 | 0.976 |
|  | Simple mode | 90 | 0.123 (-0.124, 0.370) | 0.331 | 0.662 | 39 | 0.033 (-0.148, 0.213) | 0.723 | 0.723 |
|  | Weighted median | 90 | 0.086 (-0.033, 0.204) | 0.158 | 0.316 | 39 | -0.027 (-0.139, 0.086) | 0.642 | 0.642 |
|  | Weighted mode | 90 | 0.098 (-0.072, 0.268) | 0.262 | 0.525 | 39 | -0.013 (-0.127, 0.102) | 0.832 | 0.832 |
|  | MR-PRESSO | 90 | 0.070 (-0.009-0.150) | 0.085 | - | 39 | 0.024 (-0.044-0.093) | 0.490 | - |

Note: IVW, inverse variance weighted; RE, random effect model; FE, fixed effect model; FDR, False Discovery Rate; VEGF, vascular endothelial growth factor; VEGFR, vascular endothelial growth factor receptor; SNP, single nucleotide polymorphism; β, beta; CI, confidence interval.

**Supplement Table 3**. The overlap of sample size.

| **Exposure** | | | **Outcome** | | | Overlap proportion (%) |
| --- | --- | --- | --- | --- | --- | --- |
| Variable | Source | Sample size | Variable | Source | Sample size |  |
| VEGF-A | INTERVAL | 3301 | Atrial fibrillation | >50 studies | 604990 | 0 |
|  | INTERVAL | 3301 | Atrial fibrillation and flutter | FinnGen | 218792 | 0 |
| VEGF-C | INTERVAL | 3301 | Atrial fibrillation | >50 studies | 604990 | 0 |
|  | INTERVAL | 3301 | Atrial fibrillation and flutter | FinnGen | 218792 | 0 |
| VEGF-D | 13 cohorts | 47195 | Atrial fibrillation | >50 studies | 604990 | 0.32 |
|  | 13 cohorts | 47195 | Atrial fibrillation and flutter | FinnGen | 218792 | 0 |
| VEGF (Uncategorized) | 13 cohorts | 47195 | Atrial fibrillation | >50 studies | 604990 | 0.32 |
|  | 13 cohorts | 47195 | Atrial fibrillation and flutter | FinnGen | 218792 | 0 |
| VEGFR-2 | INTERVAL | 3301 | Atrial fibrillation | >50 studies | 604990 | 0 |
|  | INTERVAL | 3301 | Atrial fibrillation and flutter | FinnGen | 218792 | 0 |
| VEGFR-3 | INTERVAL | 3301 | Atrial fibrillation | >50 studies | 604990 | 0 |
|  | INTERVAL | 3301 | Atrial fibrillation and flutter | FinnGen | 218792 | 0 |

Note: VEGF, vascular endothelial growth factor; VEGFR, vascular endothelial growth factor receptor;

INTERVAL, between mid-2012 and mid-2014, blood donors aged 18 years and older were recruited at 25 centres of England’s National Health Service Blood and Transplant.

**Supplement Table 4**. The result of modified Cochran’s Q test for heterogeneity test.

| Outcome | Exposure | Modified Cochran’s Q | | | |
| --- | --- | --- | --- | --- | --- |
|  |  | IVW | *P* | MR Egger | *P* |
| Atrial fibrillation | VEGF-A | 15.39 | 0.635 | 15.16 | 0.651 |
|  | VEGF-C | 23.30 | 0.025 | 16.15 | 0.184 |
|  | VEGF-D | 22.05 | 0.686 | 21.05 | 0.740 |
|  | VEGF (Uncategorized) | **55.59** | **1.03E-05** | **55.44** | **1.09E-05** |
|  | VEGFR-2 | 17.29 | 0.241 | 17.04 | 0.254 |
|  | VEGFR-3 | 23.77 | 0.205 | 23.57 | 0.213 |
| Atrial fibrillation and flutter | VEGF-A | 20.02 | 0.394 | 19.19 | 0.445 |
|  | VEGF-C | 19.03 | 0.088 | 17.34 | 0.137 |
|  | VEGF-D | 24.07 | 0.572 | 23.96 | 0.578 |
|  | VEGF (Uncategorized) | 21.40 | 0.26 | 20.53 | 0.304 |
|  | VEGFR-2 | 12.97 | 0.604 | 12.74 | 0.622 |
|  | VEGFR-3 | 29.63 | 0.100 | 28.59 | 0.124 |

Note: MR, mendelian randomization; VEGF, vascular endothelial growth factor; VEGFR, vascular endothelial growth factor receptor; IVW, inverse variance weighted.
